# Exploring development pathways for mining rural areas in the context of urban–rural integration: A case study in Shanxi Province, China

**DOI:** 10.1371/journal.pone.0315605

**Published:** 2025-03-10

**Authors:** Hao Xu, Shengyu Zhang, Zhenyu Wu

**Affiliations:** 1 Ankang University, Ankang, China; 2 Central China Normal University, Wuhan, China; 3 East China Jiaotong University, Nanchang, China; Qufu Normal University, CHINA

## Abstract

In the process of urban–rural integration, rural depression poses a severe challenge to urban-rural coordination and regional sustainable development. Exploring development pathways for resource-dependent rural areas is an important measure to implement rural revitalization. Mining industry, shows beneficial in boosting the economies whiles brings ecological, physical/psychological and social problems to local community members. Therefore, efforts should be made to rural sustainable development in mining resource- dependent regions. Taking the traditional mining-industry Shanxi Province in North China as research case, this paper seeks to investigate the crucial factors influencing rural transformation development (RTD) of mining regions and explore development pathways for resource-dependent rural areas. First, rural economic-labor coupling mode and rural development type of 30 mining industries-based counties were determined based on the proposed rural economic-labor elasticity coefficient, followed by the hot spot analysis for understanding the spatial correlation of different coupling modes. Then, non-parametric test was applied for feature difference test. Finally, mining rural areas transformation pathways were given by using land use transfer matrix and chord diagram. Results indicated that the mean value and rank mean value of each indicator of different rural development types were basically consistent in the distribution form, while coupling modes showed a distinct difference. This research can provide important references for rural transformation promotion, rural revitalization and sustainable rural development of resource-dependent regions, especially for developing countries.

## Introduction

Urban and rural areas are two major regional organizational units in geographical space as well as two important components in the process of social and economic development [1]. The urban-rural relationship has universal and symbiotic link, where urban and rural areas influence and restrict each other under specific social and economic conditions. However, the urban-priority development strategy has prompted a large number of production factors such as labor, land, and capital to gather in urbans, resulting in unbalanced and uncoordinated urban-rural development, which seriously restricts the sustainable development of rural areas, and causes deep-seated problems such as unsound urbanization, rural areas hollowing, prominent contradictions in land use, and environmental degradation. Therefore, many countries try to improve the urban-rural integrated development. The integrated development of urban and rural areas refers to the coordinated development and integration of urban and rural areas in economic, social, spatial and environmental aspects through a series of policies and practices. How to reshape the relationship between urban and rural areas, reconstruct rural economic development and stimulate the vitality of rural development has become an urgent problem to be solved.

As the basic industry of rural development, agriculture bears the employment and living needs of the majority of farmers. Driven by urbanization and industrialization, the flow of agricultural labor has also become the norm with the policy and economic development. With the continuous improvement of rural modernization level, the efficiency of agricultural production has risen sharply, and the number of labors absorbed by agriculture has been continuously reduced. At the same time, traditional industry, e.g., mining, has brought significant gains for local residents. However, the rural sustainable development is now facing many problems in terms of ecological and social issues, especially in resource-dependent regions. Driven by the vigorous development of secondary and tertiary industries and comparative interests, the transfer of agricultural surplus labor has been accelerated. In recent years, research on labor transfer and rural economic development has mainly focused on the impact of labor transfer on the economy [2,3], factors affecting labor transfer [4,5] and the trend of labor transfer [6]. However, there are few studies on the coupling relationship between changes in rural labor and economic development, especially for transfers between agriculture and industry. Rural transformation should be placed in the background of regional rural economy development, and the research on the labor productivity coupling mode and its featured rural development types has practical significance for classifying and promoting rural development research. Therefore, this paper explores the evolution process and spatial pattern of the coupling model between mining rural areas’ labor transfer and economic development of Shanxi Province (traditional mining industry-based sample) in North China from the scale of counties and the perspective of urban-rural integration, and deeply analyzes the types of rural development in the process of urban-rural transformation development, which provides scientific reference for promoting development of resource-dependent regions.

## Literature review

Although a considerable number of scholars have focused on rural-urban relations, rural development, and green economic growth, existing literature still exhibits deficiencies in certain crucial domains. For instance, there is an absence of systematic analysis regarding the long-term mechanism of rural-urban interaction and its influence on the transfer of rural labor, particularly in resource-dependent regions. Additionally, there is a relative scarcity of discourse on how to strike a balance between green economic growth and the coordinated development of the economic environment in less developed regions. Hence, this study endeavors to establish a comprehensive theoretical framework to systematically analyze the interactive relationship between rural labor transfer and green economic development within the context of urban-rural integration.

### Urban–rural integration

Before discussing the topic of rural development, it is necessary to emphasize the development and evolution of urban-rural relations theory and urban–rural integration. In developed countries, the theoretical research on urban–rural relations has achieved mature results. They mainly include the “Lewis-Fei-Ranis” model [7], the “Desakota” model [8], and the “regional network” model [9,10] and so on. However, these theories usually only analyze separate parts of the urban-rural system. In recent years, researchers have begun to try to solve this problem. For example, [11] discussed the relationship between urban and rural areas from the perspective of land use. [12] constructed a theoretical framework of urban-rural integration based on population, land, industry and rights; [13] discussed the policy evolution path of China’s urban-rural integration development, and adopted two approaches of new urbanization and rural revitalization to improve the quality of urban and rural development.

Before discussing the issue of rural development, the development and evolution of urban–rural relationship theory and urban–rural integration should be firstly emphasized. The theoretical research on urban-rural relations in developed countries have achieved mature results, mainly includes “Lewis-Fei-Ranis” model [7], “Desakota” model [8], “The Regional Network” model [9,10] etc. However, understanding of rural and urban land systems in that time was usually based on analysis of rural or urban systems in isolation. In recent years, many efforts were made to deal with this issue. [11] explored relation between rural and urban areas from a land use perspective.[12] constructed a theoretical framework for urban–rural integration based on population-land-industry-right between the urban and rural systems. [13] explored the policy evolution path of urban–rural integration development in China and suggested that new urbanization and rural revitalization should be adopted as two different ways to deal with urban and rural diseases and improve the quality of urban and rural development.

As a representative developing country, China has also explored the development of its own urban-rural relations. Since the implementation of economic reform in 1978, China has changed the traditional centrally planned economy into market-oriented economy, and the primary agricultural economy has been gradually shifted to an urban and rural industrial economy [14]. Meanwhile, inter-regional rural inequalities between the eastern, central and western regions have been more serious. [15] defined four rural development types and classified the rural development types in eastern coastal China, aiming for providing some implications that can promote rural development, especially for those less developed regions. From the environmental perspective, [16] investigated the effects of rural-urban development transformation on energy consumption and CO_2_ emissions on the regional and national levels.

In summary, rural transformation development should be placed in the background of urban-rural integration. In terms of research thought or research framework, studies on rural transformation development from the perspective of “model-mechanism-solution” are relatively insufficient.

### Rural development and revitalization

Rural development has been increasingly influenced by regional and urban development, coupled with the effects of globalization and informatization [17]. Current research on rural development and revitalization mainly focuses on differentiation rural development [18–20], spatial and temporal patterns for rural development [21–22]; optimization and regulation of rural revitalization paths [16,23,24]. Currently, there has been consistent conclusions that the inﬂuencing factors of rural development have experienced a process from single factor to multifactor integration. Also, the inﬂuencing factors include exogenous factors (e.g., urbanization, industrialization, and marketization) and endogenous factors (e.g., resource environment and location conditions).

Developed countries, with relatively mature technologies and sufficient capital, have promoted rural development in various ways. In the 1970s, Japan proposed and implemented one village one product (OVOP) movement to promote the development of villages by leveraging their endogenous power [25]. South Korea, another East Asia developed country, launched the new village movement (NVM) to reduce the urban-rural gap, improve rural competitiveness, and achieve a balance between urban and rural development by encouraging farmers to be self-reliant and build cooperative relationships among farmers [26]. In Europe, there have been remarkable structural changes in recent years. France implemented territorial planning during the “30 Glorious Years” period, which constructed an endogenetic and long-term rural revival mechanism aiming to solve the unbalances of territorial development [12]. England established Defra- the Department for Environment, Food and Rural Affairs to mark a new departure in the government’s treatment of rural policy [1]. Regarding the rural development theory and urban-rural integration, the United States attached great importance to the coordinated development of industry and agriculture, and promoted urban-rural integration via the “metropolitan circle model” [14].

In terms of developing countries, researches focused on shifting process and patterns from agricultural perspective to urban perspective. [27] examined land use changes in the suburbs of Bangkok by using aerial photograph interpretation and field measurements. [28] simulated future land use/cover changes up to 2030 based on a Markov-cellular automata model, results indicated that if the land use/cover trends continued in the study area without holistic sustainable development measures, severe land degradation will ensue. From multiple perspectives, [29] examined the temporal changes in 18 selected indicators of rural systems in Bangladesh form 1975–2000, and explored the influences of demographic, market forces, environmental, institutional and technological factors inducing and mediating such changes.

In summary, researches involving rural development from different point of views, understanding dynamic changes of land use, population and industry etc. However, coupling relationship between changes in rural labor and economic development are rarely discussed, leaving a significant research gap.

### Rural labor transfer of mining rural areas

Green economic growth emphasizes the coordinated development of economy and environment, while it is difficult for less developed regions to balance. For instance, mining exploitation has great influence on transfer of rural labor, the development of agriculture, and increasing the income of peasants. [30] explored the relationship between mining activities and multidimensional poverty at the municipality level in Colombia rural region by using the National Agricultural Census. Results suggested that mining with titles is not correlated with poverty levels, while mining without titles tended to increase poverty in the same municipality. [31] developed an integrated model to investigate the relationships among environmental regulation (ER), technological innovation (TI) and regional green growth performance. Findings supported the view that green growth practices may be promoted by TI driven ER, but whether ER can bring green growth practices is uncertain. [32] conducted a comprehensive evaluation of the environmental impact regarding resource-based enterprises transfer from eastern to the midwestern regions in China. Results showed that the cross-regional transfer can not only degrade the ecological environment, but also improve it, such that the resultant impact is dependent on the interaction between the increasing effect and weakening effect of environmental stress. In order to better understand the effectiveness of large-scale extractive industries and align their contribution to the sustainable development of local communities and the environment around their mining sites, [33] reviewed the literature on large-scale extractive investments in Africa published between 2000 and 2022. Findings showed that the offer of large extractive industries to communities was still low, and some were declining over time as technology advances.

## Materials and methods

Under the background of urban–rural integration, it is a complicated process to study the coupling relationship between rural labor transfer and economic development. Traditional methods such as linear regression models or simple statistical analysis, despite their advantages in capturing some basic trends, often ignore spatial heterogeneity and nonlinear relationships in urban–rural interactions. In order to better understand this complex relationship, methods such as economic-labor elasticity coefficient, hot spot analysis and non-parametric testing are used in this study. These methods show unique advantages in dealing with multiple and complex factors. The economic-labor elasticity coefficient allows us to quantify the sensitivity of the agricultural economy to changes in the labor force, especially in resource-dependent regions, which accurately reflects the economic-labor coupling. Hotspot analysis, on the other hand, provides an effective way to capture local clustering in space, which is crucial to understanding the differences between regions. At the same time, non-parametric test methods, such as Kruskal–Wallis H test, can detect significant differences between rural development types in different regions without assuming data distribution, which is particularly suitable for diversity analysis in rural transformation studies. These options address the complexity of the study more comprehensively than other approaches, especially in cases where the data do not fully conform to traditional assumptions, providing more reliable results. Therefore, the use of these methods in this study is not only innovative in theory, but also has more practical application value in empirical analysis. The selection of these methods is based on an in-depth analysis of existing research and a clear need for research questions, making the results more convincing and scientific.

### Study area

**Fig 1:**
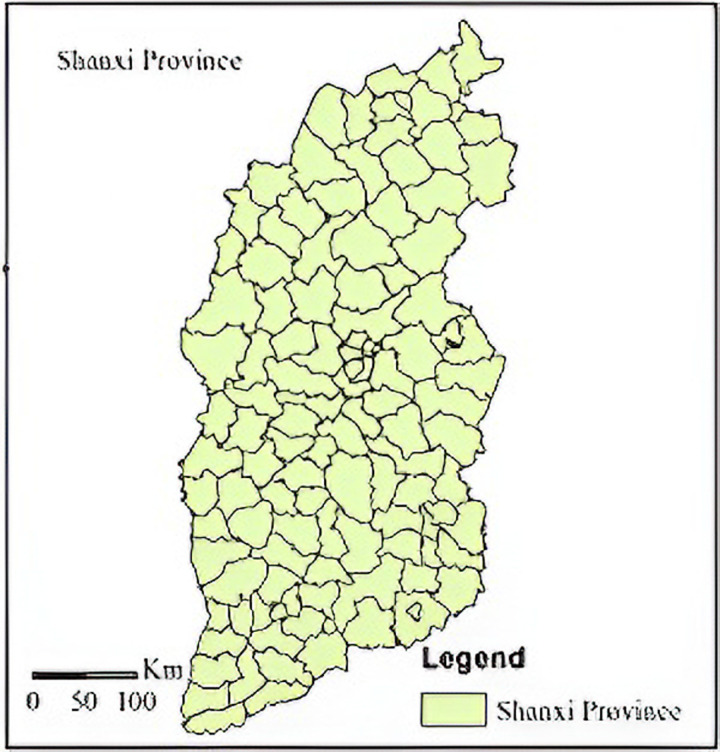
Shanxi Province, China.

As shown in Fig 1, Shanxi Province, located in North China, west of the Yellow River, characterized by its elevated terrain and a network of intersecting mountains. The region predominantly consists of plateaus and mountains, covering an area of approximately 156,700 square kilometers. Shanxi Province falls within a temperate continental climate zone, experiencing hot and rainy summers, as well as cold and dry winters, with an average annual temperature ranging from 8 to 12 °C. Renowned for its abundant coal reserves, Shanxi Province stands as a significant coal-producing area in China. The coal industry has long been a pillar of Shanxi’s economy, playing a vital role in the nation’s energy supply and industrial development. Moreover, Shanxi Province boasts a considerable expanse of arable land, favorable for crop cultivation. Agriculture holds a prominent position within Shanxi’s economy, with major crops including wheat, corn, soybeans, peanuts, and more. Fruit tree cultivation, such as apples, grapes, and apricots, is also well-known in the province. Agriculture serves as crucial components of the rural economy, providing employment opportunities and income sources for local residents. However, Shanxi Province currently faces challenges concerning rural population decline and a shortage of agricultural labor. Addressing these issues necessitates the implementation of measures to promote rural revitalization and agricultural modernization. This study focuses on Shanxi Province as the research area to investigate the influence of agricultural labor transfer on the agricultural economy amidst the context of urban-rural integration. The findings of this study aim to provide valuable scientific insights for advancing rural revitalization and transformation efforts. There has been various adjustment of administrative division, so the division in 2020 is selected. And the first 15 and last 15 GDP ranking counties are chosen as case study.

### Data sources

The empirical research in this paper takes 30 representative counties in Shanxi Province as the basic research unit. The cross-sections of time interval are 2010, 2015, and 2020. The statistical data come from the “Shanxi Statistical Yearbook” and prefecture-level city statistical yearbooks of in the corresponding years.

### Research methods

#### Analysis of economic-labor coupling mode and development type of mining rural areas.

As a crucial instrument for investigating the connection between rural economic development and labor transfer, the rural economy-labor coupling model emerged from the research on the interdependence of the urban and rural economies. Early investigations primarily concentrated on how the urban and rural economic systems operated independently. However, in recent years, with the acceleration of urban-rural integration, researchers have started to focus on the coupling dynamic relationship between rural economic development and the labor market (Pan et al., 2018; Liao et al., 2020). This model can effectively elucidate how the intricate interaction between economic activities and labor migration in resource-dependent rural areas, particularly in mining-dominated rural areas, can achieve the SDGS by coordinating economic development and labor allocation.

**Fig 2:**
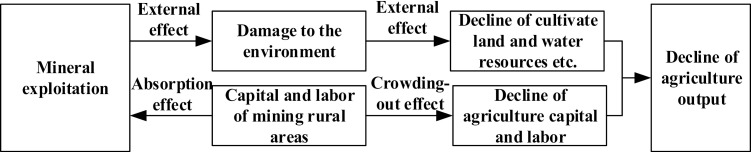
The mechanism framework of the relationship between agricultural development and mineral exploitation in mining rural areas.

Starting from the urban–rural regional system theory, the mechanism framework of the relationship between agricultural development and mineral exploitation in mining rural areas is introduced, as shown in Fig 2. The rural regional system is a spatial framework composed of humanities, economy, resources and environment, which are interconnected and interacted with each other. It not only requires to meet the needs of rural residents in terms of production, living and ecology, but also requires to guarantee the supply of agricultural products, ecological landscape and leisure space for urban residents. China has entered a rapid evolution stage of urban-rural relations, which is featured by a massive transfer of rural population to cities and a spectacular wave of seasonal migrant workers. The employment focus of the rural population has begun turning to non-agricultural industries, and a large amount of rural land has rapidly turned to urban land, which has prompted changes in the agricultural industrial structure, employment structure, and agricultural production methods, and made the rural development entering a new stage of transformation and upgrading. However, under the dual system, the long-term urban-biased development strategy, citizen-biased distribution system, and heavy industry-biased industrial structure have deepened the “three divisions” contradiction of urban division, land division and separation of people and land, which has restricted the process of the change of rural development methods and the transformation of rural development. Therefore, under the new background of urban-rural integration, urban and rural regional systems promote the re-allocation of resources and trigger the flow of factors with the influence of resource endowment, economic development, and social culture. As a result, the rural economy development and the agricultural labor are continuously developed coordinately, and the rural inner-core system is changed, thereby promoting rural transformation and reconstruction, and promoting the realization of rural revitalization. However, there are differences in natural environment, resource endowment, economic foundation, policy system, social culture, location conditions, etc. in different mining rural areas, and the types of rural development are different. Therefore, it is necessary to clarify the status quo of rural development in each region, and customize the pathways of rural revitalization according to the actual situation.

This study uses economic-labor coupling model to analyze the relationship between economic development and labor market in mining rural areas. The core of the model is to measure the ratio between the rate of change of the agricultural economy and the rate of change of the agricultural labor force in a certain period by calculating the economic-labor elasticity coefficient. This ratio reflects the degree of elasticity between the two, that is, the sensitivity of changes in the agricultural labor force to agricultural economic development.

The strength of the economic-labor coupling model is that it can provide an intuitive quantitative indicator to reveal the complex dynamic relationship between the rural economy and the labor market, especially in resource-dependent regions, such as the mining rural areas of Shanxi Province. The main advantage of the economic-labor coupling model is strong interpretation and wide applicability. This model can quantify the coupling relationship between agricultural economic development and labor market and has wide applicability and can be adapted to various data sets. However, the economic-labor coupling model also has some limitations. For example, the model relies on high-quality time series data, and the accuracy and completeness of the data have an important impact on the model results. Secondly, the application of the model may be limited by the choice of variables, especially in the case of incomplete data or lack of relevant indicators, the explanatory power of the model may be weakened.

#### Analysis of economic-labor coupling mode of mining rural areas.

Referring to the previous research results and the labor-farmland elasticity coefficient [34], the economic-labor elasticity coefficient was established to represent the ratio of the change rate of agricultural economy to the change rate of agricultural labor quantities within a certain period of time, to measure the relationship between changes in the number of agricultural labor and the development of agricultural economy in mining rural areas. The ratio can reflect the elasticity between the two sides. The larger the absolute value of the ratio, the higher the sensitivity between the two sides. On the contrary, the smaller the absolute value of the ratio, the lower the sensitivity between the two sides. It is calculated as follows:


$$ELEC_{mn}=\frac{ECR_{\text{mn}}}{LCR_{mn}}=\frac{\left(E_{\text{mn}}-E_{\text{m0}}\right)/E_{m0}}{\left(L_{mn}-L_{m0}\right)/L_{m0}}$$ (1)

Where, *ELEC*_*mn*_ is the rural economic-labor elasticity coefficient in a certain county; *ECR*_*mn*_ and *LCR*_*mn*_ represent the change rate of agricultural economic development level in region *m* in year *n* and the change rate of agricultural labor quantities, respectively; *E*_*mn*_ and *L*_*mn*_ represent the level of agricultural economic development and the number of agricultural labors in region *m* in year *n*, respectively; *E*_*m0*_ and *L*_*m0*_ respectively represent the level of agricultural economic development and the number of agricultural labor in the initial year of region *m*.

In the Cobb Douglas function, the elasticity of capital output is *α*and the elasticity of labor force is*β*. With the same conditions, capital investment of 1% increases the total output value by α%; an increase of 1% in labor input leads to an increase of β% in total output value. This can be used to predict the production of industrial systems or large enterprises in countries and regions, and analyze the mode of production development.


$$Y=Ae^{rt}K^{\alpha }L^{\beta }$$ (2)

Where, *Y* is the output, *K* is the capital, *L* is the labor, *r* is the technological progress rate.

The model takes logarithms:


lnY=lnA+rt+αlnK+βlnL (3)

This model can be used to calculate *α* and *β*

#### Coupling types of agricultural labor change and rural economics.

By analyzing the changes in the economic-labor elasticity coefficient, the coupling types of agricultural labors and rural economic development are divided into six types: growth type, extensive type, intensive type, lagging type, declining type and recession type. And we summarized and refined the coupling relationship model between the two sides and the type of rural development it represents.

#### Hot spot analysis.

Cold and hot spot analysis is a method for determining local spatial autocorrelation, which is used to measure the clustering relationship between each observation unit and the surrounding units, and to identify the cold spot area and hot spot area of the economic-labor coupling type in Shanxi Province.

Commonly employed methods and techniques for hotspot analysis include the Getis-Ord Gi * statistic, and spatial autocorrelation.

Getis-Ord Gi * statistic: This statistic is used to detect local spatial clustering and can identify hot and cold spots. It calculates the number of events in the adjacent geographic units around each geographic unit and compares it with the overall expected value.


$${\text{Gi}}^{*}\left(d\right)=\frac{X_{i}-\mu }{S\sqrt{\frac{n-1}{\sum_{j\neq i}w_{ij}}}}$$ (4)

where, $X_{i}$ is the number of events in geographic unit *i*; *μ* is the average of the total number of events; *S* is the standard deviation of the total number of events; *n* is the total number of geographical units.

This method can also evaluate the degree to which each element within a specified domain is surrounded by elements with similar high or low values.


$$Gi^{*}\left(d\right)=\frac{\sum_{j}w_{ij}\left(d\right)x_{j}}{\sum_{j}x_{j}}$$ (5)

where $Gi^{*}\left(d\right)$is the local G-statistic for a feature (i) within a distance (d), $x_{j}$ is the attribute value of each neighbor, and $w_{ij}$ is the spatial weight for the target-neighbor *i* and *j* pair.

The statistical significance of the degree of local autocorrelation between each feature and its neighbors was assessed by a z-score test. The z-scores and p-values reported for each feature, whether spatial clustering of high or low values, or spatial outliers are more pronounced than expected from a random distribution. A z-transformed form of $Gi^{*}$ by taking the statistic $Gi^{*}\left(d\right)$ minus its expectation, and reports a z-score and p-value for each single feature.


$$Gi^{*}\left(d\right)=\frac{\sum_{j}w_{ij}\left(d\right)x_{j}-\bar{X}\sum_{J}w_{ij}\left(d\right)}{s\sqrt{\frac{n\sum_{j}w_{ij}^{2}-{\left(\sum_{j}w_{ij}\left(d\right)\right)}^{2}}{n-1}}}$$ (6)

$$\bar{X}=\frac{\sum_{i}x_{j}}{n}$$ (7)

$$\text{S}=\sqrt{\frac{\sum_{\text{j}}{\text{x}}_{\text{j}}^{2}}{\text{n}}-{\left(\bar{\text{X}}\right)}^{2}}$$ (8)

Where $Gi^{*}\left(d\right)$ is computed for feature (*i*) at a distance (*d*) standardized as a z-score, ${\text{x}}_{\text{j}}$ is the attribute value of each neighbor, $w_{ij}$ is the spatial weight for the target-neighbor *i* and *j* pair and *n* is the total number of samples in the dataset.

Spatial autocorrelation: Spatial autocorrelation analysis is a method to evaluate the spatial correlation in geographic data. It can detect global and local spatial clustering patterns to help determine the distribution of hot and cold spots.


$$I=\frac{\sum_{i}\sum_{j}w_{ij}\left(X_{i}-\mu \right)\left(X_{j}-\mu \right)}{\sum_{i}{\left(X_{i}-\mu \right)}^{2}}$$ (9)

$$I=\frac{n}{S_{0}}\frac{\sum_{i=1}^{n}\sum_{j=1}^{n}W_{ij}\left(x_{i}-\mu \right)\left(x_{j}-\mu \right)}{\frac{1}{n}\sum_{i}^{n}{\left(x_{i}-\mu \right)}^{2}}$$ (10)

$$I_{i}=\frac{\left(x_{i}-\mu \right)\sum_{j=1}^{n}W_{ij}\left(x_{i}-\mu \right)}{\frac{1}{n}\sum_{i}^{n}{\left(x_{i}-\mu \right)}^{2}}.$$ (11)

where, *I* is the spatial autocorrelation coefficient, $x_{i}$ and $x_{j}$ are attributes in the i-th and j-th spaces respectively, *n* is the total number of geographical units, *u* is the average value, $W_{ij}$ is the spatial weight matrix, $S_{0}$ is the sum of all weights.

#### Non-parametric test.

In order to investigate the influence of different features on the types of mining rural areas development, this paper uses non-parametric tests to reveal the differences of independent samples. This paper mainly uses the Kruskal–Wallis H test method in the non-parametric test to verify whether there are significant differences in different features of the rural development types represented by the changes of economic-labor coupling model in different counties. The K–W test is usually used for the comparison of multiple continuous independent samples to determine whether there are differences in the overall distributions from which each sample group comes [35].

Assuming there are *k* samples, each contains a set of values. If all values are not parallel, the test statistic is:


$$\text{H}=\frac{12}{\text{N}\left(\text{N}+1\right)}\sum_{\text{i}=1}^{\text{k}}\frac{{\text{R}}_{\text{i}}^{2}}{{\text{n}}_{\text{i}}}-3\left(\text{N}+1\right).$$ (12)

Where N is the total number of values in all samples, ${\text{n}}_{\text{i}}$ is the number of values contained in the i-th sample, ${\text{R}}_{\text{i}}$ is the sum of ranks in the i-th sample.

If there is a parallel situation, it is necessary to divide H by C:


$$\text{C}=\frac{1-\sum_{\text{i}=1}^{\text{g}}\left({\text{t}}_{\text{i}}^{3}-{\text{t}}_{\text{i}}\right)}{{\text{N}}^{3}-\text{N}}.$$ (13)

Where *G* is the number of groups with bound values; *t* is the number of bound values in the i-th group.

To sum up for the case with existence of ties, the test statistic is:


$$H_{c}=\frac{H}{C}.$$ (14)

If there is no tie,C=1 and thus $H_{c}=H$

#### Land use transfer matrix.

The land use transfer matrix can effectively quantitatively study the mutual conversion between different land use/land cover types in the regional land system in a specific period, which is beneficial to express the quantitative structural features of land use in a specific period and the transfer direction between different land use types. The general form of the land use transfer matrix is:


$$S_{ij}=\left[\begin{array}{cccc} S_{11} & S_{12} & ... & S_{1n}\\ S_{21} & S_{22} & ... & S_{2n}\\ ... & ... & ... & ...\\ S_{n1} & S_{n2} & ... & S_{nn} \end{array}\right].$$ (15)

Where *S*representative area, *n* is number of land use types before and after transfer,$i.j\left(i,j=1,2,...,n\right)$ represents the land use types before and after transfer respectively, $S_{ij}$ is the area of land class *i* converted to land class *j* before transfer.

#### Chord diagram visualization.

Chord Diagram is an effective method for visually expressing the interrelationships between a large amount of complex data, and has been applied in disciplines such as biology and informatics. Therefore, in order to identify the transformation of the economic-labor coupling type in the study area in a specific period, the land use transfer matrix was used to visualize in the chord diagram, so as to construct the trajectory model of the economic-labor coupling type.

## Results

### Spatial-temporal evolution and coupling features of labor changes in mining rural areas

#### Spatial-temporal evolution of labor changes in counties.

**Fig 3:**
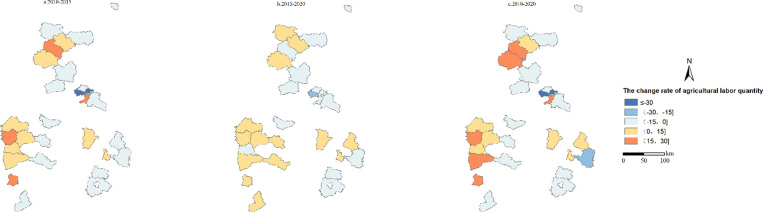
Spatial-temporal pattern of agricultural labor changes in the partial Shanxi Province counties from 2010 to 2020.

The spatial-temporal features of labor changes in partial counties of Shanxi Province are shown in Fig 3. And the rate of change is divided into high-speed decline zones, medium-speed decline zone, low-speed decline zone, low-speed growth zone, medium-speed growth zone, and high-speed growth zone according to ≤ ‒30, ( ‒30, ‒15], ( ‒15, 0], (0, 15], (15, 30], > 30, respectively. Overall, the number of labors in most counties showed a fluctuating and declining trend during the study period, with significant differences in different periods.

From 2010 to 2015, the number of agricultural labors in 60% of the selected counties in Shanxi Province was decreasing, and a large number of surplus agricultural labors were transferred. Among them, Xiaodian District, Yingze District were in the high-speed decline zone (≤ ‒30). 46.7% of the counties were in the low-speed decline zone ( ‒15, 0]. The labors in Shuocheng District, Pianguan County, Ji County, Raodu District and Yonghe County were in a low-speed growth rate (0, 15]. During the period from 2015 to 2020, the increasing rate of the agricultural labor in each county slowed down, and the number of counties with high-speed increasing were all disappeared.

**Fig 4:**
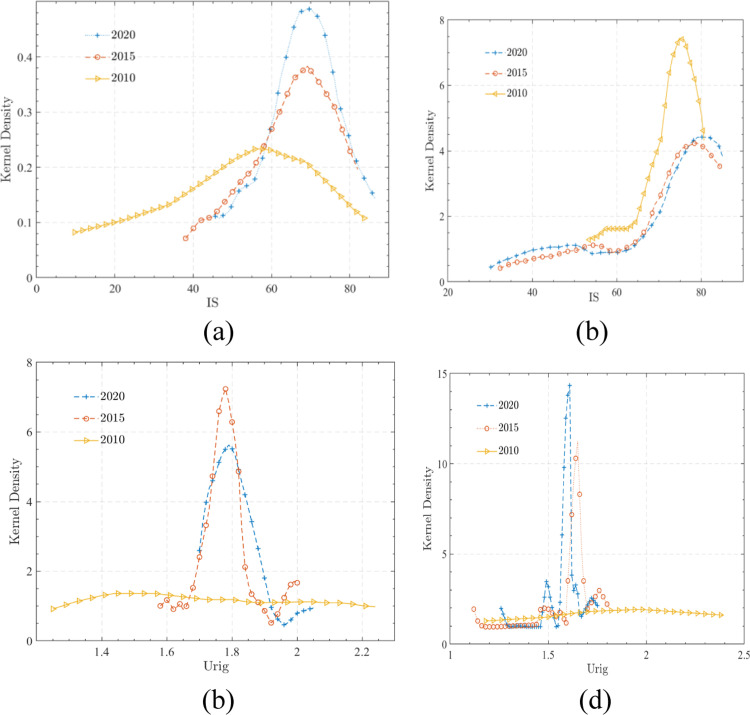
The nuclear density curve of labor force transfer and urban-rural integration in selected mining rural areas.

As shown in Fig 4, the dynamic evolution of labor transfer and urban-rural integration is demonstrated, the transfer of labor has affected the labor economy in these mining rural areas. Fig 4(a) shows the kernel density estimation curve for non-agricultural employment, the kernel density estimation curve of non-agricultural employment first shifts to the right and then to the left, indicating a trend of “first decreasing and then increasing” in non-agricultural employment from 2010 to 2020. At the same time, the peak of its nuclear density estimation curve increased from 2010 to 2020, indicating that the non-agricultural employment gap was continuously decreasing during that period. Fig 4(b) shows the kernel density estimation curve of non-agricultural output value, the center of the kernel density estimation curve of non-agricultural output value shows a rightward shift, indicating a steady increase in non-agricultural output value from 2010 to 2020. The peak height of the kernel density function shows a trend of first decreasing and then increasing, indicating that during the research period, the gap between the non-agricultural output values of various counties in Shanxi Province showed a trend of first expanding and then narrowing. Fig 4(c) shows the kernel density estimation curve of the urban-rural income gap, the center of nuclear density continues to shift to the right, with the peak height first increasing and then decreasing, indicating a trend of narrowing the urban-rural income gap between 2010 and 2020, and an absolute gap of narrowing and then expanding. Fig 4(d) shows the kernel density estimation curve of the urban-rural consumption gap, the nuclear density estimation curve shows a continuous left shift trend and the peak shows a continuous upward trend, the kernel density estimation curve shows a continuous trend of moving left and the peak shows a continuous upward trend, indicating that the absolute gap between urban and rural consumption in mining rural areas is constantly narrowing. The high value of the urban-rural consumption gap highlights the development from single polarization to multi-level, and regional imbalance still exists.

**Fig 5:**
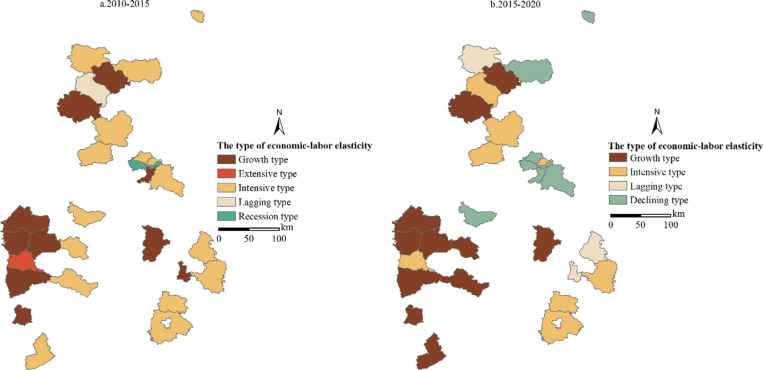
Spatial-temporal pattern of economic-labor elasticity types in partial Shanxi Province counties from 2010 to 2020.

#### Coupling features of agricultural labor changes and rural economic development.

As shown in Fig 5, the coupling models of rural economic development and agricultural labor changes in partial Shanxi Province counties show spatial and temporal differences. From 2010 to 2015, there were 16 intensive counties, accounting for 53.3%, ranking first in the coupling models of economic-labor changes in the selected mining rural areas, which were mainly distributed in most counties in the middle and east Shanxi Province. There were 9 growth-type counties, accounting for 30%, mainly distributed in the west Shanxi Province. There was only one extensive county, namely Hejin City.

From 2015 to 2020, there were only 8 intensive counties. Compared with the previous period, the scope was significantly reduced. The growth-type ranked first with a ratio of 36.7%, showing an obvious increasing trend. Meanwhile, there have been three counties where the level of economic development has declined.

### Coupling mode hot spot analysis of agricultural labor change and economic development

**Fig 6:**
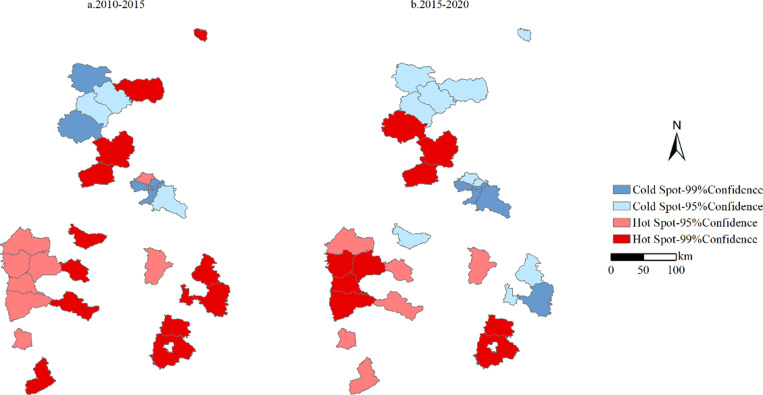
Distribution of economic-labor coupling cold and hot spots in partial Shanxi Province region from 2010 to 2020.

In order to understand the spatial correlation of the labor output coupling types in mining rural counties of Shanxi Province, the cold and hot spot analysis was used to assign values to each labor output coupling type, and the intensive type was assigned the highest value, and the declining type was assigned the lowest value. The Gi index value was divided into 4 levels according to the critical value of the Z value at the level of 1% and 5%, respectively named Hot Spot-99% Confidence, Hot Spot-95% Confidence, Cold Spot-95% Confidence and Cold Spot-99% Confidence. From this, we can get the local spatial autocorrelation diagram of labor output coupling from 2010 to 2020 (Fig 6).

#### Time series features.

From 2010 to 2020, the proportion of the number of hot spots showed a downward trend, from 39.02% to 9.76%. The proportion of sub-hot spots showed an upward trend, rising from 21.95% to 31.71%. The proportions of sub-cold spot areas and cold spot areas both showed an upward trend, rising from 21.95% and 17.07% to 26.83% and 31.71% respectively. During the period from 2010 to 2020, the labor output coupling model was dominated by hot spots, sub-hot spots, and sub-cold spots. During the period from 2015 to 2020, the number of hot spots decreased significantly, and the quantity structure mainly changed to cold spot and sub-hot spot areas.

#### Analysis of spatial features.

It can be seen from Fig 6 that the number of hotspot areas and sub-hotspot areas in Shanxi Proving almost maintains the same level. But sub-cold spot areas showed an increasing trend, from 10% to 33.3%. During the period from 2015 to 2020, the hotspot areas and sub-hot spot areas were the largest.

### The difference test of the features of development types in selected mining rural areas

“Agriculture-rural-farmers” are the three basic elements of rural development. The process of rural development is the result of the interaction of multiple factors such as population, economy, society and culture, and is the process of urban-rural population flow and economic society reorganization in the process of urban-rural integration. Theoretically, the types of rural development represented by the coupling model of agricultural labor changes and agricultural economic development should have different features in terms of agricultural production supply, rural economic development, and farmers’ production and life. Following the principles of scientific, typicality and operability, this paper constructs the following characteristic index system of rural development types: the agricultural production and supply system selects per capita farmland area, agricultural machinery level, and economic crop sowing area to reflect the differences in resource endowments and agricultural production methods among counties. Farmers’ living standards system selects farmers’ per capita disposable income and non-agricultural employment rate to reflect the employment structure features of the rural population.

This paper firstly draws the ranking chart of the mean values of various indicators in various types of mining rural counties under different systems, and observes the distribution of the mean values of characteristic indicators in various types of counties. Secondly, the non-parametric test method is used to test whether there are significant differences in the characteristic indicators of various types of counties as a whole. Finally, the rank-transformation analysis was used to compare the various types of counties in pairs to explore the specific differences, and the results were summarized Table 1. The non-parametric test results show that except for the value of non-agricultural production, the other seven indicators all pass the 5% significance test, indicating that there are significant differences in most of the characteristic indicators in various types of counties.

**Table 1:** Summary of non-parametric test.

Rural type	Variable	H-statistic	Rank mean value	Variable	H-statistic	Rank mean value
Resource dependent	Growth rate of per capita disposable income of farmers	16.826^**^	77.00^c+ d + ^	Proportion of urban construction and industrial and mining land area	155.417^**^	96.44^b + c + d-^
Modern agriculture			75.42^c+ d +^			42.22^a‒d‒^
Plant-breeding combination			55.25^a‒b-^			34.44^a‒d‒^
Industrial-transfer			35.72^a‒b-^			156.5^a + b + c + ^
Resource dependent	Non-agricultural employment rate	14.824^**^	21.05^d^‒1	Non-agricultural production value	81.179^**^	54.92^b‒c‒d‒^
Modern agriculture			31.00^d‒^			84.56^a^ ^+d‒^
Plant-breeding combination			30.17			95.50^a + d‒^
Industrial-transfer			43.13^a + b + ^			143.48^a + b + c + ^
Resource dependent	Per capita farmland area	13.604^**^	106.71^b + d + ^	Agricultural Machinery Power per Land	6.608	87.63
Modern agriculture			84.69^a‒^			96.53
Plant-breeding combination			97.03			110.44
Industrial-transfer			71.57^a-^			77.60
Resource dependent	Proportion of economic crop sowing area	15.973^**^	92.46^c‒d +^	Proportion of government expenditure to support agriculture	179.000^**^	81.50^b‒c-d + ^
Modern agriculture type			93.76^d + ^			138.50^a + c-d + ^
Plant-breeding combination			96.76^d + ^			171.50^a + b + c + ^
Industrial-transfer			67.56^a‒b‒c‒^			24.50^a‒b‒c‒^

Note:

* ,

** represent the variables pass the significance test of 5% and 1% respectively; the superscript in the rank mean value represents the difference between the type and the type represented by letters in this indicator passes a 5% significance test, such as ‘a+’ means that this indicator of the type is significantly higher than that of type 1, ‘b-’ means that this indicator of the type is significantly lower than that of type 2, and so on. Because the standard deviation of each type of variable, the proportion of government expenditure on agriculture, is 0.00, the difference can be directly judged after the non-parametric test shows that the population is not all equal.

**Fig 7:**
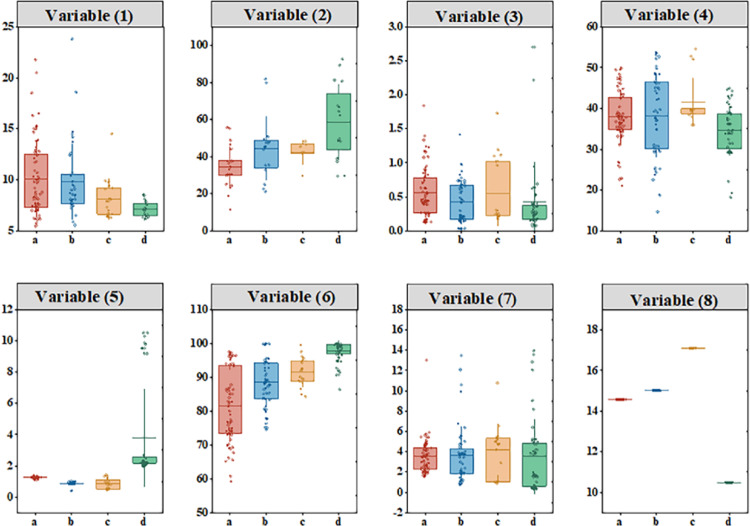
Box Graph of various variables for different rural development type of partial Shanxi Province region from 2015 to 2020.

**Fig 8:**
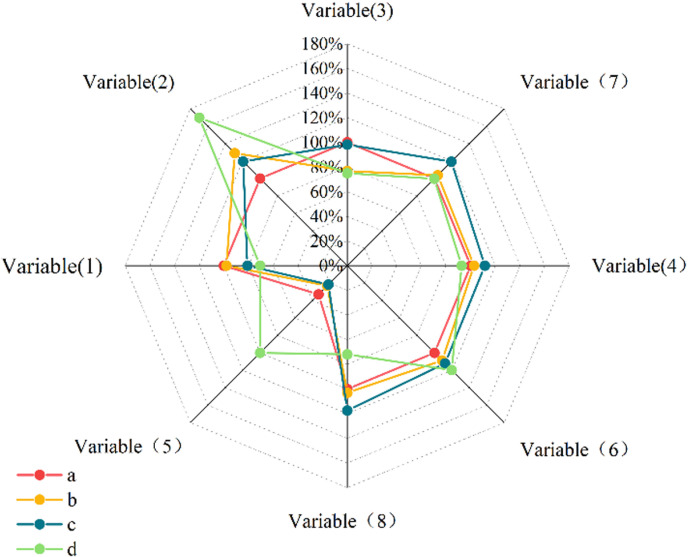
Average distribution of different characteristics of rural development types in partial Shanxi Province region from 2015 to 2020.

**Fig 9:**
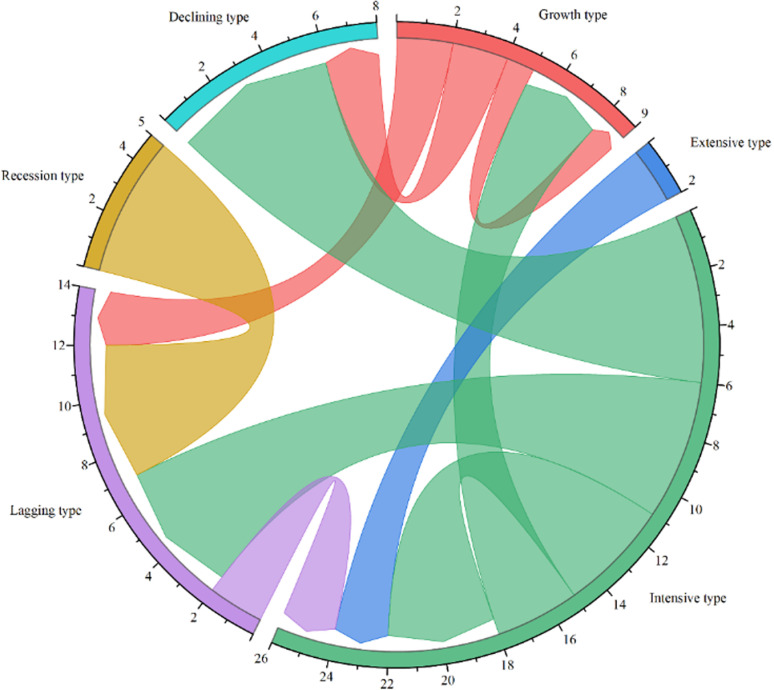
Chord chart of changes in economic-labor coupling type from 2010 to 2020 in selected mining rural areas of Shanxi Province.

In the agricultural economic system, the non-agricultural production value presents a pattern of decreasing order of resource dependent type (mining industry-based), modern agriculture type, featured agriculture type, industrial-transfer type, and plant-breeding combination type. Due to the serious non-grain conversion of cultivated land, the proportion of economic crops in the production value is similar in various types of counties. In the system of farmers’ living standards, the non-agricultural employment rate presents a pattern of decreasing order of featured agricultural type, plant-breeding combination type, resource dependent type, modern agriculture type, and industrial-transfer type. Due to the seriously transfer of agricultural labor, the non-agricultural employment rate of various types of counties is similar (Figs 7, 8, 9).

### The transformation of coupling types and pathway analysis of mining rural areas

#### The transformation of coupling types between rural labor changes and economic development.

**Fig 10:**
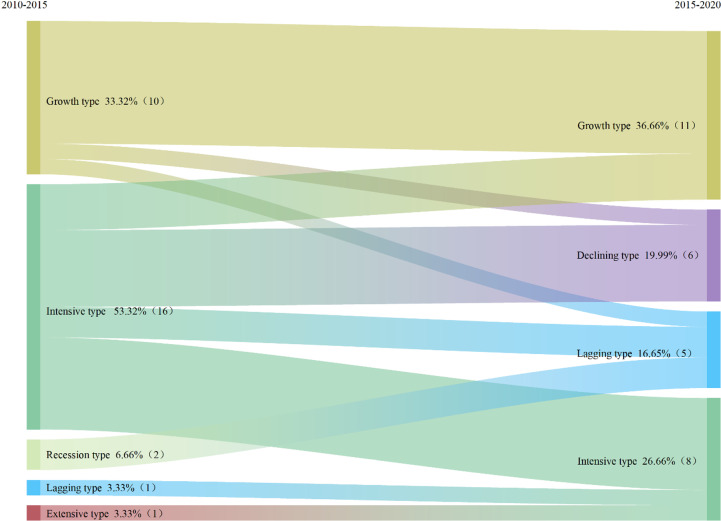
Sankey diagram of changes in economic-labor coupling type from 2010 to 2020 in selected mining rural areas of Shanxi Province.

Based on the transfer matrix and chord diagram of the economic-labor coupling mode changes during 2010 to 2015 and 2015 to 2020 (Table 2, Fig 10), and according to the rural development types from 2015 to 2020, suggestions for optimizing agricultural and mining rural areas development in counties were put forward.

**Table 2:** Coupling type in partial Shanxi Province region from 2010 to 2020.

Year	2010–2015	2015–2020
Growth type	10	11
Extensive type	1	0
Intensive type	16	8
Lagging type	1	3
Declining type	0	8
Recession type	2	0

From Table 2 and Fig 10, it can be seen that: (1) from 2015 to 2020, the growth-type counties (resource-dependent type) accounted for 19.51%, and the counties that maintained this pattern in the two periods before and after 2015 accounted for only 2.44%, which was Wangcheng district. Counties transformed from intensive type to growth type accounted for 17.07% of the counties in the study area. (2) Extensive counties (featured agricultural type) accounted for 19.51% from 2015 to 2020, and only 2.44% of counties maintained this pattern in the two periods before and after 2015, which was Xiantao City. The largest proportion of counties converted from intensive type was 17.07%. (3) From 2015 to 2020, the intensive counties (modern agricultural type) accounted for 43.90%, and 36.59% of the counties maintained this model in the two periods before and after 2015, and most of the counties maintained the original development model (Tables 3 and 4).

**Table 3:** Transfer matrix of coupling type from 2010 to 2020.

			2015–2020		
Year	Type	Growth type	Intensive type	Lagging type	Declining type
	Growth type	26.66% (8)	0.00% (0)	3.33% (1)	3.33% (1)
2010–2015	Extensive type	0.00% (0)	3.33% (1)	0.00% (0)	0.00% (0)
	Intensive type	10.00% (3)	20.00% (6)	6.66% (2)	16.66% (5)
	Lagging type	0.00% (0)	3.33% (1)	0.00% (0)	0.00% (0)
	Recession type	0.00% (0)	0.00% (0)	6.66% (2)	0.00% (0)

**Table 4:** Coupling type change process and trends in partial Shanxi Province region during 2010 to 2020.

Index	Growth type	Extensive type	Intensive type	Lagging type	Declining type	Recession type
N_c_ (%)	10%	0%	‒50%	200%	0%	‒100%
T_c_ (%)	50%	0%	75%	600%	0%	100%
P_s_	0.2	0	‒0.66	0.33	0	‒1

#### Pathway analysis of mining rural areas transformation.

**Fig 11:**
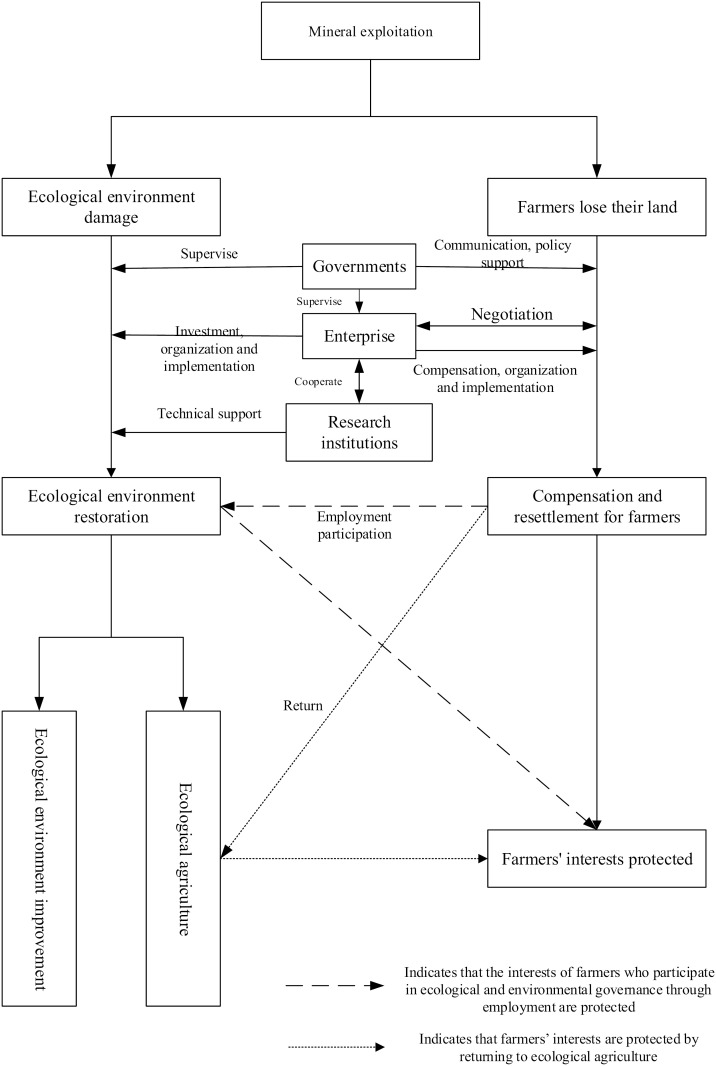
Pathway for mining rural areas transformation.

From the perspective of mining industry, industry transformation and diversification have always been the key point, which can avoid excessive economic fluctuation. In terms of case study in this research, Shanxi Province also has the advantage of tourism, equipment manufacturing and biological medicine industry except for mining resource. Efforts should be made to expanding the proportion of manufacturing and service industries in Shanxi’s economy, providing employment opportunities and promoting the transfer of rural labor. Fig 11 gives an innovation idea for mining rural areas transformation.

Another way is to proper develop coal-related industries such as coking, electric power and coal chemical industry and try to realize new-type orientation of traditional industries. Shanxi is also the main production area of aluminum, iron and other resources. The government should develop electrolytic aluminum and aluminum processing industry, and develop High-end steel industry relying on the Taiyuan Iron & Steel Co., Ltd. (TiSCO) Industrial Park. Shanxi has accumulated rich experience in coal logistics, coal service industry, coal industry technology innovation by energy base construction, so it should actively realize the transformation from coal mining industry to coal service industry.

From the perspective of agricultural, resource dependent counties have obvious dependence on the development of agricultural economy. Strategies for this type should focus on industrial structure transfer and new types of business entities. For example, the development of the agricultural product processing industry can be accelerated to adjust and optimize the agricultural structure and improve the quality of agricultural products, so as to achieve high-quality development of the agricultural structure. The agricultural economic growth rate of featured agricultural type counties is slower than the increase of agricultural labor, and the agricultural production efficiency of its agricultural labor is low. Therefore, it is necessary to take advantage of regional advantages, clarify regional positioning, actively innovate new agricultural formats, promote the development of leisure agriculture and rural tourism, and at the same time increase government support to cultivate advantageous and featured agricultural industry clusters. The level of urbanization and agricultural modernization development in agricultural modern counties is relatively high, which provides the basis for labor transfer and agricultural economic growth. Based on the advantages of urbanization and industrialization, this type of counties should continue to promote the integration of the primary, secondary and tertiary industries of agriculture, encourage the expansion of multiple functions of agriculture, and tap the multiple values of rural areas, focus on the development of agricultural product processing, rural leisure tourism, and rural e-commerce industries, and promote modern agricultural industrial parks. Due to the downward trend of the agricultural economy, there occurs labor transfer in industrial transfer counties because of the serious loss of labor and the rise of secondary and tertiary industries. Therefore, the government should strengthen infrastructure construction and capital investment. At the same time, for some areas with serious labor loss, corresponding policies should be introduced to attract talents to return to their hometowns, to promote local employment and entrepreneurship, to implement various support policies and vigorously carry out skills training, new occupation and new business training suitable for farmers’ employment. Due to the prominent agricultural diversity and small geographical scale, the agricultural industries are clustered and dispersed. Therefore, for scattered and diverse industries, it is necessary to take advantage of favorable regional advantages, implement differentiated development for different “agricultural conditions”, strengthen in-depth industrial development, and establish local characteristic brands to realize the effective aggregation and optimal allocation of agricultural production factors, and promote the continuous, steady and rapid advancement of agricultural modernization. In promoting the strategy of rural revitalization, it is necessary to design differentiated rural development policies based on the development models of various villages.

## Conclusions

In the context of urban-rural integration, this paper reveals the spatial-temporal evolution and coupling features of labor changes and economic development in mining rural areas of Shanxi Province through the economic-labor elasticity coefficient and coupling features, and analyzes the significant difference of various rural types through the non-parametric test. Finally, the rural revitalization pathway is proposed. The main research conclusions are as follows:

From 2010 to 2015, most counties in Shanxi Province were in the high-speed growth range. The growth rate of agricultural economy during 2015-2020 was lower than that of the previous research period. The high-speed growth areas have been greatly reduced, and only 35.89% of the counties have maintained the original growth rate. The coupling mode of agricultural economic development and labor changes in counties showed spatial-temporal differences. From 2010 to 2015, the intensive counties ranked first in the changes of economic-labor coupling mode. Compared with the previous period, the scope of intensive counties from 2015 to 2020 has shrunk significantly, but it still ranked first with a proportion of 43.90%.

From 2010 to 2020, the proportion of hot spots showed a downward trend, the proportion of sub-hot spots showed an upward trend, and the proportions of sub-cold spots and cold spots areas both showed an upward trend. During 2010–2020, the annual economic-labor coupling mode was dominated by hot spots, sub-hot spots, and sub-cold spots. From 2015 to 2020, the number of hot spots areas decreased significantly, and the quantity structure changed to be dominated by cold spots and sub-hot spots. The hot spot, sub-hot spot, cold spot, sub-cold spot area changed from “multi-core agglomeration” and point-like distribution coexistence to “single-core agglomeration”, from “dual-core agglomeration” to “band-like distribution”, from “two-core agglomeration” to “single-core agglomeration” and “point-like distribution” coexistence, and from “single-core agglomeration” to the spatial distribution pattern of “multi-core agglomeration”.

Through the non-parametric test, it is found that the mean value and the rank mean value of each indicator of the rural development type in each county are basically consistent in the distribution form. In the agricultural production and supply system, the area occupied by urban construction and industrial and mining land shows a decreasing trend of industrial-transfer type, featured agricultural type, resource dependent type, plant-breeding combination type, and modern agriculture type. The non-agricultural production value presents a pattern of decreasing order of resource dependent type, modern agriculture type, featured agricultural type, industrial-transfer type, and plant-breeding combination type. In the system of farmers’ living standards, the non-agricultural employment rate presents a pattern of decreasing order of featured agricultural type, plant-breeding combination type, resource-dependent type, modern agriculture type, and industrial-transfer type.

The development of mining rural region transformation is not a simple one-way linear development process, but interacts with cities in the process of urbanization and industrialization to form a stable urban-rural complementary relationship, thereby promoting the optimization and reconstruction of rural production systems, living systems, and ecological systems. The factors affecting rural transformation are complex and changeable, and these factors can affect the transformation development of the entire countryside individually or through the interaction between factors. Due to the complexity of the rural system and the features of regional rural development, the influencing factors and effects of the rural system in different regions and different scales are quite different. In this paper, the county is taken as the basic unit, and the selection of factors affecting rural development features are mostly dominant by resource endowment and mining industrial features. It mainly explores the impact of common features on different types of mining rural region development. However, at a smaller microscopic scale, changes in farmers’ livelihoods methods, such as engaging in rural tourism and planting featured agricultural products, have an impact on the transformation. Analyzing the influencing factors and internal mechanism of mining rural region development from a micro perspective needs to be discussed in follow-up research.

This study not only provides a new perspective for understanding the coupling relationship between rural labor transfer and economic development in mining areas, but also provides empirical evidence for policy makers to help them formulate more accurate rural development policies. However, considering the complexity and limitations of the research, future studies can further explore the changes in farmers’ livelihood patterns at the micro level and their impact on rural economic transformation. In addition, the conclusion of this study can be verified by comparative study of other resource-dependent areas, and the theory of rural development can be further improved.

## Supporting information

S1 Data GDP Rank top, 15. (XLSX)
